# Exogenous DCPTA Ameliorates Simulated Drought Conditions by Improving the Growth and Photosynthetic Capacity of Maize Seedlings

**DOI:** 10.1038/s41598-017-12977-1

**Published:** 2017-10-04

**Authors:** Tenglong Xie, Wanrong Gu, Yao Meng, Jing Li, Lijie Li, Yongchao Wang, Danyang Qu, Shi Wei

**Affiliations:** 1 College of Agriculture, Northeast Agricultural University, Harbin, 150030 P.R. China; 2 The Observation Experiment Station of the Ministry of Agriculture for Crop Cultivation Science in Northeast Area, Harbin, 150030 P.R. China; 3 Heilongjiang Academy of Land Reclamation Sciences, Harbin, 150030 P.R. China

## Abstract

Previous reports have indicated that 2-(3,4-dichlorophenoxy)triethylamine (DCPTA) can promote the growth and photosynthetic capacity of plants. However, only a small number of these studies have focused on crops, and few reports have focused on whether DCPTA affects stress tolerance. In this study, maize (*Zea mays* L.) seedlings were pretreated with or without DCPTA and then exposed to drought stress in a controlled growth room for 7 days, and the growth and photosynthesis indexes of the seedlings were investigated. The DCPTA treatment partly counteracted the observed decreases in biomass, net photosynthetic rate (*Pn*), stomatal conductance (*Gs*), transpiration rate (*Tr*), effective photochemical efficiency of photosystem II (*ΦPSII*), maximum photochemical efficiency of *PSII* (*Fv/Fm*), non-photochemical quenching (*NPQ*), and photosynthetic pigment content and increased the minimal fluorescence (*Fo*) induced by drought stress. The DCPTA treatment also alleviated the damage induced by drought stress in the photosynthetic apparatus. In addition, DCPTA pretreatment simultaneously increased the root size (e.g., the length, surface area, and volume) and root hydraulic conductivity, which promoted the maintenance of higher relative leaf water contents (RLWCs) under stress conditions. These results indicate that exogenous DCPTA ameliorates simulated drought conditions by improving the growth and photosynthetic capacity of maize seedlings.

## Introduction

Maize (*Zea mays* L.), a typical C_4_ plant of the NADP-malic enzyme (NADP-ME) type^1^, represents the third-most important crop in the world after rice and wheat, and its cultivation yields ~1 billion tons of the crop annually. During its life cycle of 80~110 days, maize requires relatively large amounts of water (500~800 mm) compared with other crops. Additionally, maize is considered sensitive to drought stress, especially during critical periods of plant development, such as the seedling stage. However, maize is primarily grown in regions that are susceptible to drought, including North and South America, China and other parts of Asia, Africa and Europe^2^.

In NADP-ME-type C_4_ species, atmospheric CO_2_ is primarily fixed into oxaloacetate through the carboxylation of phosphoenolpyruvate via phosphoenolpyruvate carboxylase (PEPCase) in mesophyll cells, and then oxaloacetate is transported to the mesophyll cell chloroplasts. NADP-dependent malate dehydrogenase (NADP-MDH) reduces most of the oxaloacetate to malate, which is subsequently exported to the bundle sheath cell chloroplasts, where it is decarboxylated by NADP-ME to supply both CO_2_ and reducing power. The released CO_2_ is re-fixed by ribulose-1,5-bisphosphate carboxylase (RuBPCase) during the Calvin-Benson cycle, and the other product of decarboxylation, pyruvate, is returned to the mesophyll cell chloroplasts, where it is phosphorylated by pyruvate orthophosphate dikinase (PPDK) to regenerate phosphoenolpyruvate^3^.

Drought is known to influence an array of biochemical and physiological processes at the molecular, cellular, and whole plant levels^4^ and cause reduced plant growth and diminished crop productivity in many areas, which poses a serious challenge to food security^5–7^. Importantly, photosynthesis, one of the key metabolic processes in plants, is involved in regulating plant responses to drought stress^8^. The light reactions (in which light energy is converted into ATP and NADPH and oxygen is released) and dark reactions (in which CO_2_ is fixed into carbohydrates by utilizing the products of light reactions, ATP and NADPH) of photosynthesis occur in the chloroplast^9^, which is highly sensitive to drought and plays a prominent role in the modulation of stress responses^10^. Photosynthetic pigments are believed to be damaged by drought, which results in the reduced light-absorbing efficiency of both photosystems (*PSI* and *PSII*) and hence a reduced light reaction capacity^11^. Photosynthetic inhibition has been primarily associated with stomatal limitations during early drought stress. Stomatal closure is the most efficient method of preventing water loss under drought stress. However, stomatal closure also restricts CO_2_ diffusion into the leaves, which ultimately inhibits carbon assimilation metabolism during dark reactions^12^. In addition, root absorbance may decrease because of the reduced transpiration that occurs under drought stress^13^, and the activities of enzymes related to the Calvin cycle have been found to be inhibited by drought stress^14^.

Various agronomic and physiological practices are applied to minimize the adverse effects of drought stress on plant growth. The exogenous application of plant growth regulators has been considered an effective method of enhancing plant drought tolerance during crop production^15^. The tertiary amine bioregulator 2-(3,4-dichlorophenoxy) triethylamine (DCPTA) has been reported to increase the dry weight of leaves, stems, and roots in tomatoes^16^, increase the root development and leaf areas of radishes and beets^17^, and increase the chloroplast volume and ribulose-1,5-bisphosphate activity in the mature leaves of sugar beets^18^. This compound also promotes CO_2_ fixation in cotton^19^, accelerates seedling growth and enhances chlorophyll biosynthesis in blue spruce^20^ and guayule^21^, and stimulates carotenoid biosynthesis in citrus^22^. Although multiple studies have shown that DCPTA treatments can promote growth, its specific performance and underlying mechanisms with regard to crops are poorly understood. The performance of DCPTA on plants under non-stressed conditions has been investigated for only a few plant species, with only a small number of these studies focusing on crops. In addition, the effects of DCPTA on plant drought tolerance are poorly comprehended. Therefore, the present study was performed to investigate the effects and underlying mechanisms of DCPTA on maize under non-stressed and simulated drought conditions.

## Results

### Effects of the PEG-6000 and DCPTA treatments on seedling growth and relative leaf water content

Under non-stressed conditions, DCPTA promoted seedling growth, an effect that was more pronounced at 25 mg/L (Table 1). As the polyethylene glycol (PEG)-6000 level increased, the growth parameters of the maize seedlings reduced gradually (Table 2). The simulated drought conditions induced by the 15% PEG-6000 treatment significantly inhibited seedling growth; however, the decrease in growth was partially recovered by DCPTA, and this effect was more pronounced at 15 mg/L. Compared with the control, the shoot fresh weight, root fresh weight, shoot dry weight, root dry weight and total leaf area on the 7^th^ day increased by 8.90%, 8.88%, 5.75%, 13.46% and 8.20% in the 25 mg/L DCPTA treatment group, respectively; decreased by 45.50%, 39.80%, 37.93%, 32.69% and 28.90% in the 15% PEG-6000 treatment group, respectively; and decreased by 30.99%, 23.44%, 25.28%, 15.38% and 19.97% in the 15% PEG-6000+15 mg/L DCPTA treatment group, respectively.

**Table 1:** Effects of DCPTA (5, 15, and 25 mg/L) with or without 15% PEG-6000-induced drought stress on the fresh and dry weights of the shoots and roots and the leaf area. The values represent the mean ± SE (n = 5). Values with the same letters in the columns are not significantly different at P < 0.05 (LSD test).

Treatment	Fresh weight (g·plant^−1^)		Dry weight (g·plant^−1^)		Leaf area (m^2^·plant^−1^)
	Shoot	Root	Shoot	Root	
Control	2.123 ± 0.105c	0.691 ± 0.009b	0.174 ± 0.005b	0.052 ± 0.007b	39.480 ± 1.300b
5 mg/L DCPTA	2.188 ± 0.068bc	0.712 ± 0.018b	0.179 ± 0.007ab	0.054 ± 0.005ab	40.984 ± 1.695ab
15 mg/L DCPTA	2.241 ± 0.082ab	0.723 ± 0.009ab	0.180 ± 0.003ab	0.055 ± 0.004ab	41.504 ± 1.968a
25 mg/L DCPTA	2.312 ± 0.070a	0.753 ± 0.029a	0.184 ± 0.009a	0.059 ± 0.004a	42.718 ± 2.576a
15% PEG-6000	1.157 ± 0.066 f	0.416 ± 0.019e	0.108 ± 0.006e	0.035 ± 0.003d	28.070 ± 0.088e
15% PEG-6000+5 mg/L DCPTA	1.284 ± 0.101e	0.463 ± 0.022d	0.118 ± 0.008d	0.039 ± 0.003 cd	28.914 ± 0.817de
15% PEG-6000+15 mg/L DCPTA	1.465 ± 0.050d	0.529 ± 0.028c	0.130 ± 0.007c	0.044 ± 0.005c	31.594 ± 0.351c
15% PEG-6000+25 mg/L DCPTA	1.381 ± 0.033de	0.505 ± 0.045c	0.126 ± 0.009 cd	0.042 ± 0.004c	30.648 ± 1.423 cd

**Table 2:** Effects of PEG-6000 (10%, 11%, 12%, 13%, 14%, and 15%) on the fresh and dry weights of the shoots and roots and the leaf area. The values represent the mean ± SE (n = 5). Values with the same letters in the columns are not significantly different at P < 0.05 (LSD test).

Treatment	Fresh weight (g·plant^−1^)		Dry weight(g·plant^−1^)		Leaf area(cm^2^·plant^−1^)
	Shoot	Root	Shoot	Root	
10% PEG-6000	1.493 ± 0.069a	0.527 ± 0.020a	0.138 ± 0.006a	0.042 ± 0.006a	33.318 ± 0.319a
11% PEG-6000	1.471 ± 0.050ab	0.524 ± 0.010a	0.133 ± 0.004b	0.041 ± 0.004ab	31.570 ± 0.382b
12% PEG-6000	1.408 ± 0.050bc	0.485 ± 0.013b	0.126 ± 0.004c	0.037 ± 0.006ab	31.040 ± 0.912b
13% PEG-6000	1.334 ± 0.078c	0.457 ± 0.017c	0.116 ± 0.002d	0.037 ± 0.004ab	29.748 ± 0.554c
14% PEG-6000	1.209 ± 0.058d	0.426 ± 0.008d	0.114 ± 0.004de	0.036 ± 0.004b	28.718 ± 0.603d
15% PEG-6000	1.157 ± 0.066d	0.416 ± 0.019d	0.108 ± 0.006e	0.035 ± 0.003b	28.070 ± 0.088d

**Figure 1:**
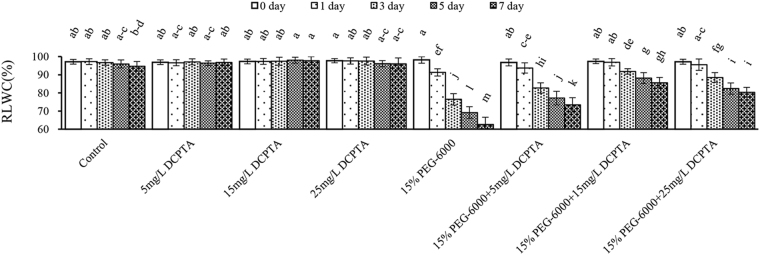
Effects of DCPTA (5, 15, and 25 mg/L) on the RLWC of the fully developed second leaf (numbered basipetally) of maize seedlings under simulated drought stress. The data represent the means of independent measurements with five replicates, and standard deviations are indicated by the vertical error bars. Values with the same letters on the bars are not significantly different at P < 0.05.

Under non-stressed conditions, the DCPTA treatment did not have a significant effect on the relative leaf water content (RLWC) (Fig. 1). On the 1^st^ day of 15% PEG-6000-induced drought stress, the RLWC decreased marginally and reached 91.26% in the control seedlings. However, compared with the control, the value decreased significantly to 76.43%, 69.13%, and 62.58% on the 3^rd^, 5^th^, and 7^th^ days, respectively (Fig. 1). The DCPTA-treated plants (15 and 25 mg/L) showed statistically significant increases in the RLWC compared with the non-DCPTA-treated plants under 15% PEG-6000-simulated drought conditions from the 1^st^ day. Moreover, there were no significant differences in the 15% PEG-6000+15 mg/L DCPTA and 15% PEG-6000+25 mg/L DCPTA treatments between the 5^th^ day and 7^th^ day. The RLWC increased by 1.17-fold, 1.37-fold and 1.28-fold in the presence of 5 mg/L, 15 mg/L, and 25 mg/L DCPTA, respectively, under the 15% PEG-6000-simulated drought conditions compared with the 15% PEG-6000-only treatment on the 7^th^ day (Fig. 1).

### Effect of the PEG-6000 and DCPTA treatments on the root architecture and root hydraulic conductivity

In the 25 mg/L DCPTA treatment group under non-stressed conditions, the maximum total root length, root surface and area root volume values increased by 9.30%, 11.23%, and 13.04%, respectively, compared with the corresponding values of the control (Table 3). The 15% PEG-6000-only drought stress treatment had a severe and deleterious impact on the total root length, root surface and area root volume, showing reductions of 43.92%, 52.82% and 45.65%, respectively, compared with the control. However, the DCPTA treatment significantly attenuated the root growth reduction induced by 15% PEG-6000. The effect of 15 mg/L DCPTA was more pronounced than those of 5 mg/L and 25 mg/L under the 15% PEG-6000-simulated drought conditions, with the total root length, root surface and area root volume values increased by 29.04%, 34.39% and 36.00%, respectively, compared with the non-DCPTA-treated seedlings.

**Table 3:** Effects of DCPTA (5, 15, and 25 mg/L) with or without 15% PEG-6000-indued drought stress on the root morphological traits of the maize seedlings on the 7^th^ day. The values represent the mean ± SE (n = 5). Values with the same letters in the columns are not significantly different at P < 0.05 (LSD test).

Treatment	Total length (cm)	Total surface area (cm^2^)	Total volume (cm^3^)
Control	513.91 ± 21.59b	50.314 ± 2.26c	0.46 ± 0.02c
5 mg/L DCPTA	540.86 ± 17.88a	53.052 ± 2.35bc	0.49 ± 0.02b
15 mg/L DCPTA	557.62 ± 24.47a	55.430 ± 2.02ab	0.53 ± 0.02a
25 mg/L DCPTA	561.72 ± 15.86a	55.964 ± 2.75a	0.52 ± 0.01a
15% PEG-6000	288.22 ± 18.78e	23.738 ± 1.83 f	0.25 ± 0.02 f
15% PEG-6000+5 mg/L DCPTA	340.98 ± 21.63d	28.238 ± 2.46e	0.30 ± 0.02e
15% PEG-6000+15 mg/L DCPTA	371.91 ± 18.30c	31.902 ± 2.16d	0.34 ± 0.02d
15% PEG-6000+25 mg/L DCPTA	358.58 ± 23.31 cd	29.024 ± 1.96de	0.32 ± 0.03de

**Figure 2:**
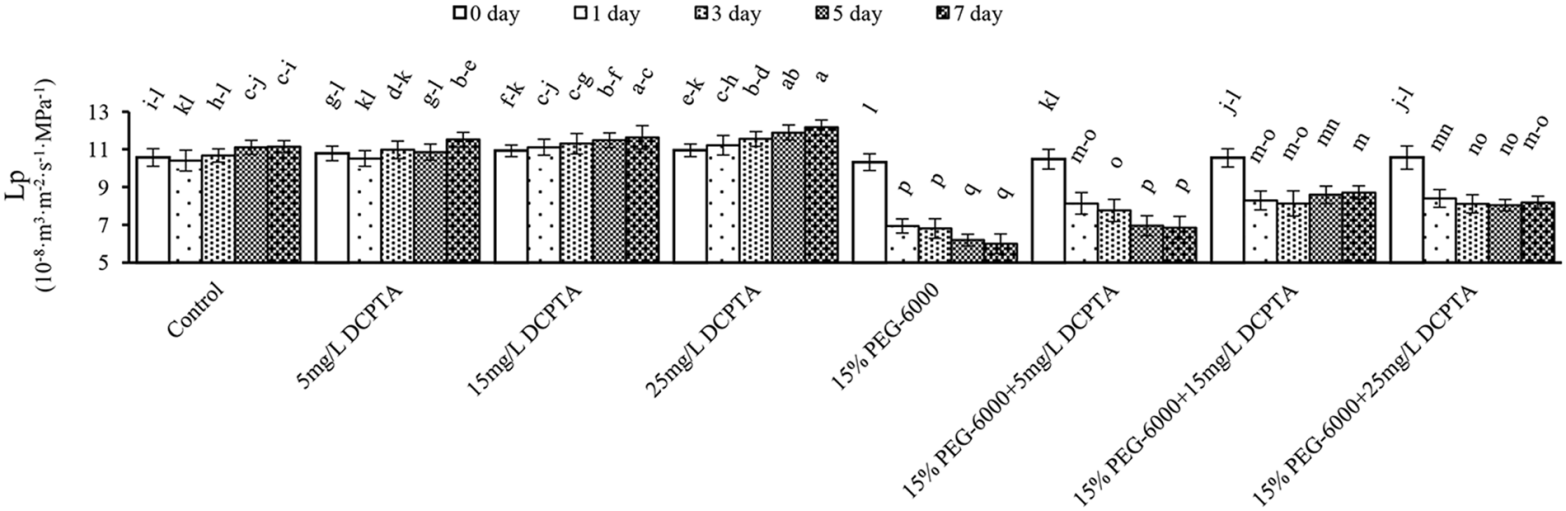
Effects of DCPTA (5, 15, and 25 mg/L) on the hydraulic conductance (Lp) of maize seedlings under simulated drought stress. The data represent the means of independent measurements with five replicates, and the standard deviations are indicated by the vertical error bars. Values with the same letters on the bars are not significantly different at P < 0.05.

Under non-stressed conditions, the DCPTA pretreatment increased the root hydraulic conductivity (Lp) over the 7-day experimental period (Fig. 2). As the 15% PEG-6000-induced drought stress progressed, the Lp decreased consistently. The Lp decreased on the 1^st^ day and 2^nd^ day of the 15% PEG-6000+DCPTA treatments and then slightly decreased in the 15% PEG-6000+5 mg/L DCPTA treatment, exhibited a minor increasing trend in 15% PEG-6000+15 mg/L DCPTA treatment, and remained constant in 15% PEG-6000+25 mg/L DCPTA treatment.

### Effect of the PEG-6000 and DCPTA treatments on the photosynthetic pigment content

Under non-stressed conditions, the DCPTA treatments (5, 15, and 25 mg/L) markedly increased the chlorophyll a (*Chl a*) content by 2.12%~8.13%, the *Chl b* content by 0.38%~8.81%, the *Chl a*+*b* content by 1.47%~8.46%, and the carotenoid (*Car*) content by 5.60%~7.80%. By contrast, the *Chl a*/*b* ratio did not show a significant change with respect to the DCPTA treatments (Table 4). Compared with the control seedlings under 15% PEG-6000 simulated drought conditions, the photosynthetic pigment contents were lower, and the *Chl a*/*b* ratios were higher. DCPTA was effective in overcoming the reduction in these photosynthetic pigments, and the optimal concentration was 15 mg/L. Following this treatment, the *Chl a*, *Chl b*, *Chl a*+*b* and *Car* contents increased by 1.43-fold, 1.67-fold, 1.53-fold and 1.13-fold, respectively, compared with the values under the 15% PEG-6000 treatment conditions, and a lower *Chl a*/*b* ratio was observed on the 7^th^ day (Table 4).

**Table 4:** Effects of DCPTA (5, 15, and 25 mg/L) with or without 15% PEG-6000-induced drought stress on the photosynthetic pigment content (mg/g FW) of the maize seedlings. The values represent the mean ± SE. Values with the same letters in a column are not significantly different at P < 0.05 (LSD test).

Treatment	*Chl a*	*Chl b*	*Chl a+b*	*Chl a/b*	*Car*
Control	2.83 ± 0.07b	2.61 ± 0.13c	5.44 ± 0.18c	1.09 ± 0.04d	2.31 ± 0.08b
5 mg/L DCPTA	2.89 ± 0.06b	2.62 ± 0.08bc	5.52 ± 0.10bc	1.10 ± 0.04d	2.44 ± 0.17ab
15 mg/L DCPTA	2.94 ± 0.08ab	2.74 ± 0.06ab	5.68 ± 0.07ab	1.07 ± 0.05d	2.46 ± 0.16a
25 mg/L DCPTA	3.06 ± 0.12a	2.84 ± 0.14a	5.90 ± 0.22a	1.08 ± 0.05d	2.49 ± 0.12a
15% PEG-6000	1.66 ± 0.09e	1.21 ± 0.05 f	2.87 ± 0.14 g	1.37 ± 0.03a	1.60 ± 0.10e
15% PEG-6000+5 mg/L DCPTA	2.16 ± 0.13d	1.72 ± 0.13e	3.88 ± 0.24 f	1.26 ± 0.06b	1.73 ± 0.08de
15% PEG-6000+15 mg/L DCPTA	2.37 ± 0.10c	2.02 ± 0.09d	4.39 ± 0.15d	1.17 ± 0.06b	1.89 ± 0.12c
15% PEG-6000+25 mg/L DCPTA	2.33 ± 0.13c	1.80 ± 0.09e	4.13 ± 0.20e	1.29 ± 0.06c	1.84 ± 0.08 cd

### Effect of the PEG-6000 and DCPTA treatments on the chlorophyll fluorescence parameters

**Figure 3:**
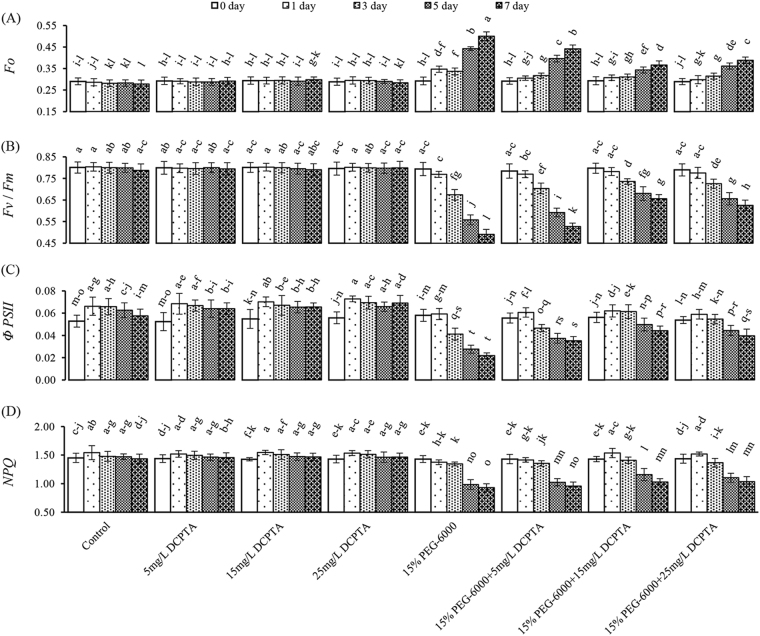
Effects of DCPTA (5, 15, and 25 mg/L) on the *F*o, *F*v/*F*m, *NPQ* and *ΦPSII* of the fully developed second leaf (numbered basipetally) of maize seedlings under simulated drought stress. The data represent the means of independent measurements with five replicates, and the standard deviations are indicated by the vertical error bars. Values with the same letters on the bars are not significantly different at P < 0.05.

The DCPTA treatment did not influence the obtained chlorophyll fluorescence parameters under non-stressed conditions (Fig. 3). Conversely, the DCPTA treatment significantly moderated the simulated drought-induced increases in the minimal fluorescence (*F*o) and decreases in the maximal quantum yield of PSII photochemistry (*F*v/*F*m), effective PSII quantum yield (*ΦPSII*) and non-photochemical quenching coefficient (*NPQ*). Compared with the 15% PEG-6000 treatment on the 7^th^ day, the DCPTA treatments (5, 15, and 25 mg/L) markedly decreased the *F*o by 11.65%~26.78% and increased the *F*v/*F*m by 7.33%~21.85%, the *ΦPSII* by 61.46~102.75%, and the *NPQ* by 2.79%~11.04%. Overall, the DCPTA at 15 mg/L had a higher positive effect on chlorophyll fluorescence parameters than the other DCPTA concentrations under the 15% PEG-6000-simulated drought conditions (Fig. 3).

### Effect of the PEG-6000 and DCPTA treatments on gas exchange parameters

**Figure 4:**
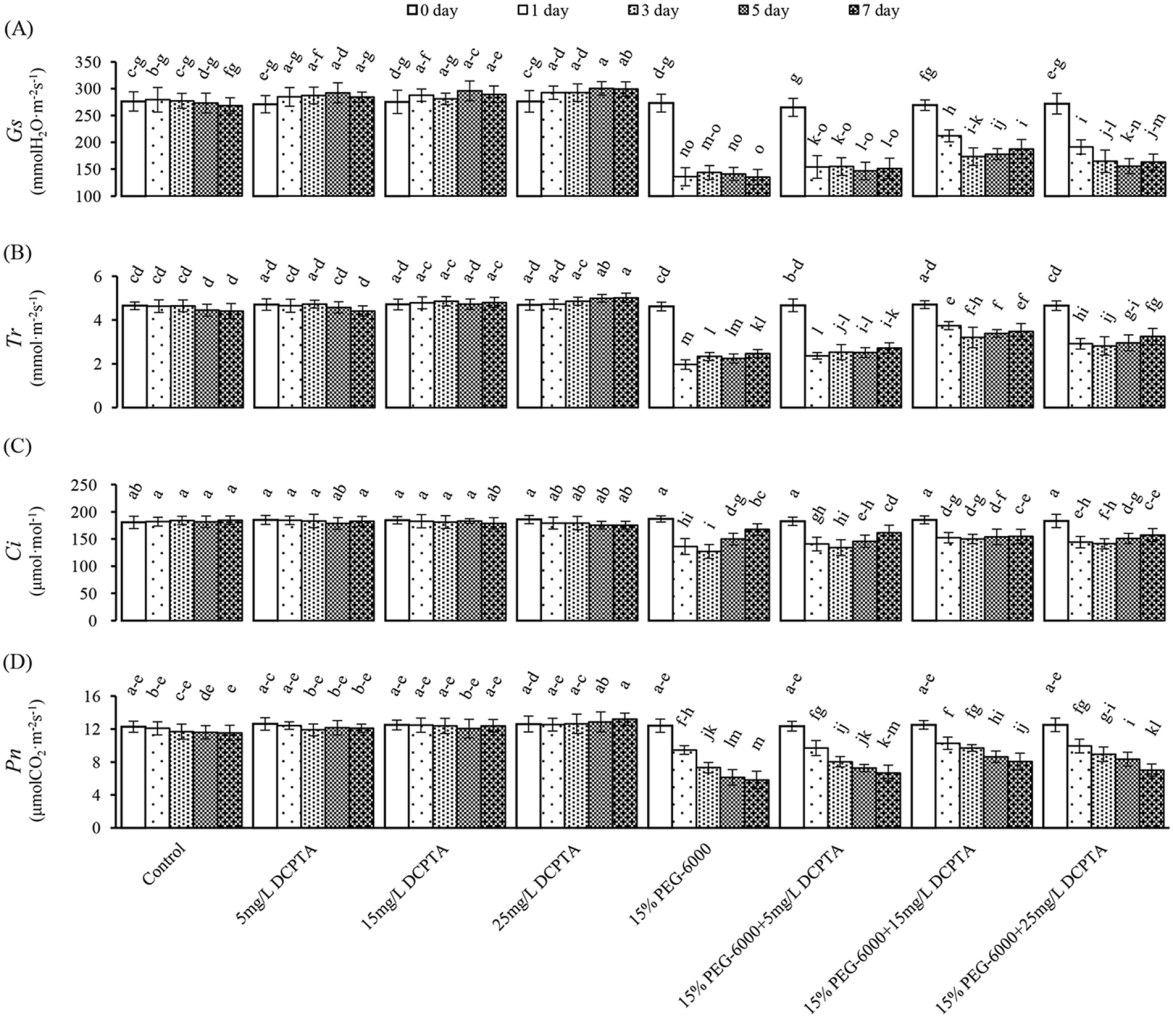
Effects of DCPTA (5, 15, and 25 mg/L) on the *Gs*, *Tr*, *Ci* and *Pn* of the fully developed second leaf (numbered basipetally) of maize seedlings under simulated drought stress. The data represent the means of independent measurements with five replicates, and the standard deviations are indicated by the vertical error bars. Values with the same letters on the bars are not significantly different at P < 0.05.

**Figure 5:**
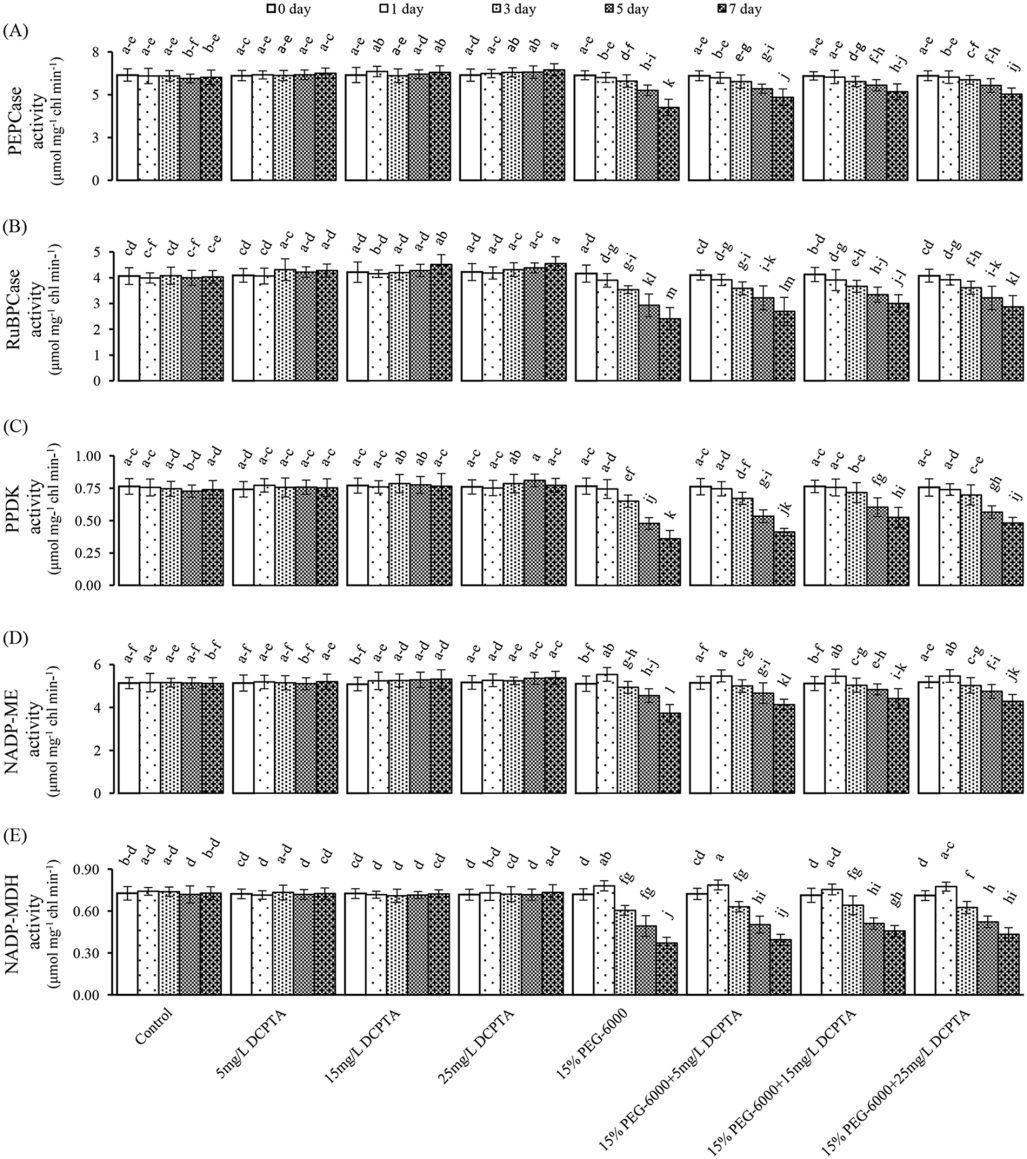
Effects of DCPTA (5, 15, and 25 mg/L) on the activities of the RuBPCase, PEPCase, PPDK, NADP-MDH and NADP-ME extracted from the fully developed second leaf (numbered basipetally) of maize seedlings under simulated drought stress. The data represent the means of independent measurements with five replicates, and the standard deviations are indicated by the vertical error bars. Values with the same letters on the bars are not significantly different at P < 0.05.

Under non-stressed conditions, the 15 and 25 mg/L DCPTA treatments had a significant effect on transpiration rate (*Tr*) and stomatal conductance (*Gs*) on the 5^th^ and 7^th^ day, whereas only the 25 mg/L DCPTA treatment increased the net photosynthetic rate (*Pn*) on the 5^th^ and 7^th^ day (Fig. 4). Furthermore, the 25 mg/L DCPTA treatment led to a considerable increase in the *Pn* (1.14-fold), *Gs* (1.11-fold) and *Tr* (1.14-fold) on the 7^th^ day compared with the control. All the parameters decreased on the 1^st^ day of the 15% PEG-6000 treatment, and the *Gs* and *Tr* values exhibited minor increasing trends thereafter. The *Pn* value continued to decrease, whereas the intercellular CO_2_ concentration (*Ci*) initially decreased and then increased during the simulated drought period. The decrease in *Pn* was partly reversed by applying DCPTA. Similarly, the *Ci*, *Gs* and *Tr* values were higher in the DCPTA-treated maize seedlings than in the untreated ones under 15% PEG-6000 simulated drought conditions, and the positive effect of DCPTA was most pronounced in the 15 mg/L treatment group. Compared with the control on the 7^th^ day, the *Pn*, *G*s, *Tr* and *Ci* values decreased by 49.61%, 49.71%, 43.92% and 9.18% in the 15% PEG-6000 treatment group, respectively, and by 30.10%, 30.26%, 21.01% and 15.99% in the 15% PEG-6000+15 mg/L DCPTA treatment group, respectively (Fig. 5).

### Effect of the PEG-6000 and DCPTA treatments on the photosynthetic enzyme activity

Over the 7-day experimental period, compared with the control, all the measured activities of the enzymes did not significantly increase, except for the activity of ribulose-1,5-bisphosphate carboxylase/oxygenase (RuBPCase) in 15 mg/L DCPTA treatment group and the activities of phosphoenolpyruvate carboxylase (PEPCase) and RuBPCase in the 25 mg/L DCPTA treatment on the 7^th^ day, as well as the activity of pyruvate phosphate dikinase (PPDK) in the 25 mg/L DCPTA treatment group on the 5^th^ day under non-stressed conditions (Fig. 5). On the 7^th^ day of the 15% PEG-6000 treatment, the PEPCase, RuBPCase, PPDK, NADP-malic enzyme (NADP-ME), and NADP-malate dehydrogenase (NADP-MDH) activities were significantly decreased by 29.28%, 40.34%, 37.75%, 27.11% and 49.04%, respectively, relative to the control. DCPTA application ameliorated the decreases in all the measured enzymes’ activities during the 7-day experimental period; moreover, all the measured enzymes’ activities in the 15% PEG-6000+15 mg/L DCPTA and 15% PEG-6000+25 mg/L DCPTA treatment groups were significantly higher than those in the 15% PEG-6000 treatment group on the 7^th^ day. On the 7^th^ day, the PEPC, RUBPC, PPDK, NADP-ME and NADP-MDH activities in the 15% PEG-6000+15 mg/L DCPTA treatment group increased by 1.21-fold, 1.25-fold, 1.46-fold, 1.18-fold and 1.23-fold, respectively, compared with those in the 15% PEG-6000 treatment group (Fig. 5).

### Effect of the PEG-6000 and DCPTA treatments on the chloroplast ultrastructure

**Figure 6:**
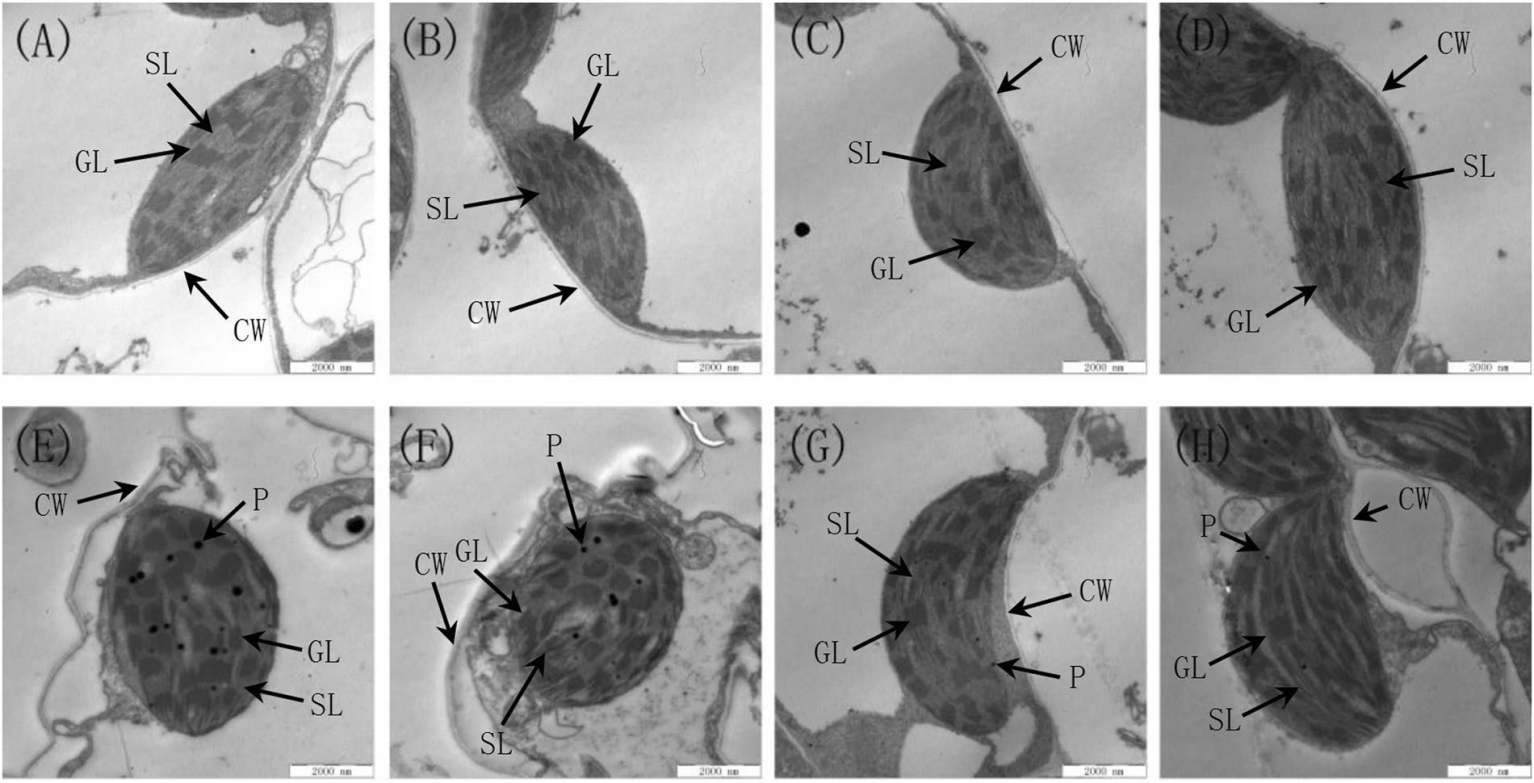
Effects of exogenous DCPTA on the ultrastructure of the photosynthetic apparatus of the leaves of maize seedlings grown in nutrient solutions with or without 15% PEG-6000. The second leaves (numbered basipetally) were sampled for ultramicroscopic observations on day 7 after the drought treatment (15% PEG-60000). SL, stroma lamella; GL, grana lamellae; CW, cell wall; and P, plastoglobule. The scale bars for the photosynthetic apparatus represent 2000 nm. (**A**) Control, (**B**) 5 mg/L DCPTA, (**C**) 15 mg/L DCPTA, (**D**) 25 mg/L DCPTA, (**E**) PEG-6000, (**F**) PEG-6000+5 mg/L DCPTA, (**G**) PEG-6000+15 mg/L DCPTA, and (**H**) PEG-6000+25 mg/L DCPTA.

Under non-stressed conditions, the chloroplasts in the seedlings were elongated ellipses that contained well-arranged grana and smooth thylakoid membranes regardless of the presence or absence of DCPTA (Fig. 6A–D). After 7 days of 15% PEG-6000-induced drought stress, the chloroplasts were nearly round and asymmetrically swollen and had an increased number and size of plastoglobules plastoglobules (Fig. 6E). Far from the cell wall, the chloroplast envelope was partially ruptured, and the thylakoid membranes were loose and disrupted, whereas the thylakoids were overly disorganized. The DCPTA treatment restored the connection between the chloroplasts and cell membranes in the simulated drought-stressed maize seedling leaves, and the shape of the chloroplasts changed slightly from elongated ellipses to ellipses (Fig. 6F–H). A well-aligned internal lamellar system and reduced number of plastoglobules were observed in the leaves subjected to the 15% PEG-6000+15 mg/L DCPTA treatment compared with those subjected to the 15% PEG-6000-only treatment (Fig. 6G).

## Discussion

PEG is a polymer penetrant that has been widely applied for the artificial creation of controlled drought conditions. Because of its convenience and operability, PEG-6000 at different levels was applied to mimic different degrees of drought stress^23^. In this study, we investigated the biomass of maize seedlings that were grown under increasing degrees of simulated drought stress (10%~15% PEG-6000). The growth inhibition induced by the simulated drought stress in this study has previously been observed in maize^24^ and other plant species^5^. The reduction in the root biomass may have arisen from root respiratory inefficiency^25^, and the reductions in the shoot biomass have been reported reductions in chlorophyll content, *PSII* function, transpiration rates and stomatal conductance as well as by the diminished activities of key enzymes involved in carbon dioxide fixation^24,26^, and these effects were also observed in the present study. Interestingly, the deleterious effects of the simulated drought conditions on maize seedling growth were partly counteracted by the DCPTA pretreatment (Table 1). For the 15% PEG-6000+15 mg/L DCPTA treatment group, the root fresh weight and dry weight were similar to those in the 10% PEG-6000 treatment group, and the leaf fresh weight, dry weight and leaf area were close to those of the 11% PEG-6000 treatment group (Tables 1 and 2). This result further implied that exogenous DCPTA ameliorates the growth inhibition effects of 15% PEG-6000-simulated drought conditions. The growth-promoting effect of DCPTA at concentrations ranging from 1 mg/L to 40 mg/L has been observed in soybeans, radishes, cotton, sugar beets, and tomatoes^27^. Interestingly, the highest concentration of applied DCPTA (25 mg/L) had the strongest positive impact on maize performance (in terms of most of the traits studied) under non-stressed conditions, whereas the most effective concentration of DCPTA in alleviating 15% PEG-6000-induced drought stress was 15 mg/L instead of 5 mg/L or 25 mg/L DCPTA (Table 1).

Because the assimilates generated via photosynthesis are major building blocks and energy sources for biomass production and maintenance^28^, greater biomass may be associated with greater photosynthetic capacity and an appropriate water status^29^, as observed for the DCPTA treatment under simulated drought conditions. The regulation of stomatal movement plays an important role in controlling gas exchange and balancing water requirements, and the maintenance of an appropriate leaf water status is an efficient adaptive mechanism for plant growth under drought conditions^30^. Several studies have shown that stomatal closure, the earliest response to drought stress, reduced the water loss and simultaneously limited ambient CO_2_ diffusion to the site of carboxylation, which is usually considered the main reason for the decline in photosynthetic rate under drought conditions^31^. The RLWC may represent a potential indicator of the plant water status^32^. In this study, the RLWC continued to decline during the 15% PEG-6000 treatment, which may have been due to an imbalance between transpirational loss and water uptake by the roots during this period, and the resulting loss of turgor may have limited the cell expansion and reduced the growth in the crop plants^33^. Although there is still a consistent decrease in this value as the stress progresses in the 15% PEG-6000+DCPTA treatment groups, the reduction in the RLWC was less in the leaves of DCPTA-treated plants under 15% PEG-6000-simulated drought conditions from the 1^st^ day (Fig. 1). This result suggested that DCPTA partly reversed the adverse effect of 15% PEG-6000-induced drought stress on the RLWC.

Root hydraulic conductance (Lp) is an important parameter that reflects the ability to uptake water and determines water movement rate throughout the whole plant^34^. In this study, the extent of the significant decrease in Lp induced by PEG-6000-simulated drought stress was diminished via the application of DCPTA from the 1^st^ day, and the *Gs* displayed similar tendency as the Lp (Figs 2 and 4A). One possible explanation for the high RLWC and *Gs* values in the stressed seedlings treated with DCPTA in our study is that the DCPTA treatments promoted water absorption, which is largely achieved by improving the root growth (which is associated with the root fresh weight, dry weight, length, surface area and volume) and root hydraulic conductance (Fig. 2). The increased *Gs* contributed to the increase in CO_2_ in the cellular spaces of the leaf and promoted the higher *Tr* value, which reduced the leaf epidermal resistance and promoted the mass flow of water to the leaf surface, as well as the transportation of substances required to conduct photosynthesis in leaves. These results showed that DCPTA alleviated the drought stress-induced inhibition of photosynthesis caused by stomatal limitation. The influence of DCPTA on *Gs* might be regarded as one of the initiating steps in the mechanism underlying the ameliorating effects of DCPTA on crops under simulated drought conditions^35^. Our findings indicated that applying DCPTA improves maize root growth and had the strongest influence on *Gs* compared to the other parameters under non-stressed conditions, which is consistent with the results of previous studies conducted with tomatoes^16^, radishes and beets^17^. The extensive root system induced by DCPTA can contribute to an increase in the nutrient and water uptake, thereby enabling plants to perform well under non-stressed conditions.

*Chl a* and *Chl b*, the primary light-absorbing and transmitting pigments (antenna pigments), can increase the light capture efficiency, which in turn can enhance the light reactions^36^. Our results suggested that the *Chl a*, *Chl b* and *Chl a*+*b* contents were significantly decreased by the 15% PEG-6000 treatment, which implies that the simulated drought conditions lead to decreased light harvesting. The decrease in chlorophyll content under simulated drought conditions may be attributed to the oxidation of chlorophyll molecules^37^ and lipids included in membranes, particularly thylakoid membranes, which causes the loss of their integrity, as detected in this study (Fig. 6E). However, the DCPTA treatments alleviated the decrease in *Chl a*, *Chl b*, and *Chl a*+*b* induced by the simulated drought stress; thus, these treatments may improve the quantum harvesting efficiency of leaves, which would lead to enhanced photosynthesis under simulated drought conditions. A possible correlation between the DCPTA application and the rise in chlorophyll contents is suggested, which could play a promising role in protecting photosynthetic proteins from oxidative damage under stress conditions^38^. The DCPTA treatment also increased the *Chl a*, *Chl b*, and *Chl a*+*b* contents in the maize seedlings under non-stressed conditions in this study (Table 4), which was also observed in blue spruce^20^. In the present study, simulated drought stress induced a decrease in the *Car* contents. The DCPTA treatment stimulated *Car* biosynthesis in the seedlings under non-stressed conditions, an effect that was also detected in citrus in a previous study^22^. Moreover, the DCPTA treatment increased the accumulation of *Car* in the simulated drought-stressed maize seedlings, thereby contributing to a dissipation of excess excitation energy, which is beneficial to the photoprotection effect of photosynthesis^39^.

In this study, we observed that the DCPTA treatment significantly diminished the decreases in the *Fv/Fm* and *NPQ* during the simulated drought period. This finding, along with the higher *ΦPSII*, implies higher electron transport to carbon fixation, which would lead to increases in the CO_2_ assimilation rate^40^. The increased *NPQ* caused by DCPTA could provide protection against damage from excessive energy under simulated drought conditions^41^. Drought stress inhibits *PSII* electron transport, and limiting the electron transfers from the reaction centre of *PSII* to the primary acceptor plastoquinone (*QA*) and the secondary acceptor plastoquinone (*QB*) inhibits the transfer of excitation energy from the light-harvesting complex (*LHC*) to *PSII* ^42^. *LHC* is the most abundant protein complexes on the thylakoid membrane, 50% of which is composed of *Chl a* and *Chl b*. Therefore, maintaining the chlorophyll content and thylakoid integrity using DCPTA under 15% PEG-6000-induced drought stress may enhance the electron transport of *PSII*. In the current study, the thylakoids became swollen, and distorted stroma and grana lamella were observed in the chloroplasts under simulated drought conditions. These findings are consistent with those reported by Shao *et al*. (2016)^43^. In addition, these results imply a synchrony between membrane degradation and plastoglobulus formation. Increased numbers of plastoglobules have been observed in the leaves of diverse plant species under various abiotic and biotic stresses, such as drought stress^44^. Austin *et al*. (2006) proposed that the lipid molecules contained in the plastoglobules are in a dynamic equilibrium with those located in the thylakoid membrane^45^. In this study, the lipids that were ejected from the plane of the membrane may be the main contributor to the formation of plastoglobules under simulated drought stress. We also observed that the cellular structure of the leaves in the DCPTA treatment groups remained intact and showed orderly chloroplasts, flattened stacks of thylakoids and a few small plastoglobules scattered in the stroma. These results imply that the DCPTA treatment improved the integrity of the chloroplast membrane structure under simulated drought conditions. The relative stability of the photosynthetic membrane structure enhanced chlorophyll synthesis and normal metabolism of *PSII* under simulated drought conditions. The results for the chlorophyll content and fluorescence parameters also support this finding. Previously, the application of DCPTA to seedlings has been reported to increase the chloroplast volume^18^; however, in this study, an increased chloroplast volume was not observed in the DCPTA-treated seedlings under non-stressed conditions.

Photosynthetic parameters (*Fv/Fm*, *ΦPSII*, *NPQ*, *Pn*, *Gs*, *Tr*, and *Ci*) were decreased already after the 1^st^ day of simulated drought, and the lower *Pn* observed in the PEG-only stress treatment was accompanied by a significant decrease in the *Ci* on the 1^st^ and 3^rd^ days. By contrast, the activities of the photosynthetic C_4_ enzymes did not significantly change on the 1^st^ day, except for slight increases in the activities of NADP-ME and NADP-MDH. This may be a reaction to maintain the rate of carboxylation under decreased CO_2_ concentration in mesophyll cells as a result of stomatal closure at the early period of drought treatment. The increased activities NADP-ME and NADP-MDH on the 1^st^ day of the PEG-6000-only treatment could represent an adaptation to drought. This result suggested that the reduction in the *Pn* was partly caused at first by stomatal limitations, whereas the photosynthetic C_4_-enzymes’ activities were affected significantly from the 3^rd^ day. This result implies that the carboxylation process was severely affected and that the decreased activities of the photosynthetic C_4_-enzymes were another major cause of the restricted photosynthesis under simulated drought conditions. When the stomata were closed and photosynthesis was reduced under drought stress, the amount of CO_2_ captured by PEPCase could be important. The increase in PEPCase activity caused by DCPTA helped maintain the rate of carboxylation under the decreased CO_2_ concentrations resulting from stomatal closure^46^. DCPTA increased the Rubisco activity of the maize seedlings under non-stressed conditions, and Keithly, J *et al*. (1990) have drawn similar inferences while working with sugar beets^18^. Moreover, DCPTA exerts similar effects under simulated drought conditions. The increased PPDK activity contributes to the rapid formation of PEP, thereby accelerating the PEP carboxylation reaction in direct proportion to the increase in PPDK^47^. The DCPTA application increased the activities of RuBPCase, PEPCase, PPDK, NADP-MDH and NADP-ME, and CO_2_ was effectively assimilated. This assimilation caused the seedlings to maintain higher carbon assimilation efficiency, which helped to improve the photosynthetic efficiency and ultimately increased the biomass under simulated drought conditions.

## Conclusion

In summary, we performed physiological and metabolic analyses of plants that were pretreated with DCPTA under non-stressed and simulated drought conditions, and the results demonstrated that DCPTA could promote seedling growth. Several mechanisms may be involved, including the following: (1) the DCPTA treatments maintained higher contents of leaf water, chlorophyll, and carotenoids and greater photosynthetic C_4_-enzyme activities, thereby resulting in maize seedlings with enhanced photosynthetic capacity and improved tolerance to simulated drought; (2) the DCPTA treatment promoted water uptake by improving root growth (in terms of the root fresh weight, dry weight, length, surface area and volume) and root hydraulic conductivity, thereby maintaining an appropriate water status; (3) the application of exogenous DCPTA minimized the damage to the chloroplasts caused by the simulated drought conditions; (4) DCPTA displayed weak effects at higher concentrations, and the optimized positive impacts of the DCPTA treatments were obtained at doses of 15 mg/L and 25 mg/L under simulated drought and non-stressed conditions, respectively, in this study. Furthermore, DCPTA also improved the maize performance under non-stressed conditions.

## Materials and Methods

### Plant material and treatments

This hydroponic experiment was conducted in a controlled growth room at the Northeast Agricultural University of Harbin, China. DCPTA was provided by the China Zhengzhou Zhengshi Chemical Limited Company. Certified disease-free maize (*Zea mays* L.) seeds of the inbred line Chang 7-2 procured from the Henan Academy of Agricultural Sciences in China were used. The maize seeds were surface sterilized for 15 min with 0.2% HgCl_2_, rinsed abundantly with distilled water, and germinated on paper towels moistened with distilled water at 28 °C for 72 h in the dark. Relatively uniform seedlings were selected and inserted into holes in styrofoam boards that had been placed in opaque plastic containers (inner length, 50 cm; width, 30 cm; and height, 18 cm) containing 10 L of 1/2 Hoagland’s nutrient solution. The experimental units were placed under the following environmental conditions: average day/night temperature of 25/18 °C, relative humidity of 65 ± 5%, light intensity of 350 μmol/m^2^/s and photoperiod of 16 h.

Uniform maize seedlings at the three-leaf stage were used for the treatments in the different nutrition solutions. Here, the study consists of two parts: 1) addition of 1/2 Hoagland’s nutrient solution supplemented with or without added DCPTA (5, 15, or 25 mg/L) to the hydroponic solution for 24 h, followed by exposure to 1/2 Hoagland’s nutrient solution containing 15% PEG-6000; and 2) 1/2 Hoagland’s nutrient solution containing 10%, 11%, 12%, 13%, 14%, and 15% PEG-6000.

The treatments were organized according to a completely randomized design (CRD) with five replicates. The nutrient solution was replaced every day to maintain the component concentration at the target value. The pH of the nutrient solution was adjusted to 6.30 (±0.05) every day. All treatment solutions were continuously aerated.

### Sampling and measurement of different parameters

The seedlings were randomly collected on the 7^th^ day after stress to determine their biomass, leaf area, and root morphological traits and to perform a microscopic examination of the chloroplasts. The fully developed second leaf (numbered basipetally) of each plant was sampled to determine the RLWC, gas exchange parameters, and chlorophyll fluorescence parameters. On days 0, 1, 3, 5 and 7 after the stress treatment, the fully developed second leaves (numbered basipetally) were excised from five seedlings from one of the opaque plastic containers of one treatment at 10:30~11:00 a.m., which represented one sample, and then were immediately frozen in liquid nitrogen and stored at −80 °C for various analyses. Each treatment had five independent biological replications.

### Biomass (fresh weights and dry weights of the shoots and roots) and leaf area

The seedlings were separated into roots and shoots, oven dried at 105 °C for 30 min, and then held at 80 °C for 24 h. The total leaf area was measured using a Li-3000 leaf area metre (Li-Cor, Inc., Lincoln, NE, USA). Average values of 10 plants from one dish were considered one replicate.

### Relative leaf water content (RLWC)

After the fresh weight was determined, the samples were soaked in distilled water for 24 h, and the surface water was removed with absorbent paper before the saturation fresh weight was measured. The samples were then dried at 105 °C for 15 min and then held at 80 °C until reaching a constant weight, which was recorded. The calculation of the RLWC of the leaf tissue was based on Munné-Bosch and others (2003)^48^.

### Root morphological traits and hydrostatic root hydraulic conductivity root activity

The root morphological traits (length, surface area, and volume) were measured using the WinRHIZO Image Analysis system (Version 2013e) (Regent Instruments Inc., Canada). The hydrostatic root hydraulic conductivity (Lp) was measured with a Scholander pressure chamber according to the method described by López-Pérez *et al*. (2007)^49^. The aerial parts of the plants were removed, and the stems were placed in plastic tubes. The roots were placed in a pressure chamber in water, and the pressure (0.2, 0.3 and 0.4 MPa) applied to the detached roots was gradually increased. Each pressure interval was maintained for 2 min, and the sap exuded during each pressure interval was collected in Eppendorf tubes and weighed. The roots and the tubes were weighed in a precision balance. The sap flow (Jv) was expressed in mg H_2_O (g root DW)^−1^ h^−1^ and plotted against the pressure (MPa), with the slope representing the L value in mg H_2_O (g root DW)^−1^ MPa^−1^ h^−1^.

### Pigment contents

The pigment contents were determined according to Arnon (1949)^50^. A frozen leaf sample weighing 0.1 g was cut into pieces and placed in a 15 mL centrifuge tube together with 10 mL of miscible liquids comprising a 1:1 mixture of 95.5% acetone and absolute ethyl alcohol. The tubes were then covered with a black plastic bag and kept in a dark place until the sample turned white. To determine the *Car*, *Chl a*, *Chl b*, and *Chl a*+*b* contents, 1 mL of the filtered extract was diluted with 6 mL of absolute ethanol, and the absorbances were measured at 470 nm, 645 nm, 652 nm and 663 nm.

### Transmission electron microscopy of the chloroplasts

The leaf samples were cut into pieces of approximately 1 mm^2^ and fixed overnight with 4% glutaraldehyde in 0.1 M phosphate-buffered saline (PBS, pH 7.4). The fixed samples were washed three times with the same solution for 10 min each. The samples were then post-fixed in 1% osmium tetroxide in cacodylate buffer for 2 h and then washed three times in 0.1 M PBS (pH 7.4). Next, the samples were dehydrated in a graded ethanol series (50%, 70%, 90%, and 100%) and absolute acetone for 15 min. The samples were embedded in Spurr’s resin, and ultrathin sections were cut and stained with uranium acetate and lead citrate in series. The ultrathin sections were mounted on copper grids and examined under a HITACHI HT7700 transmission electron microscope.

### Gas exchange parameters

The *Pn*, *Tr*, *Gs*, and *Ci* values were measured with a calibrated portable LI-6400 gas exchange system (*Li-6400, Li-Cor Inc*., USA) according to the manufacturer’s instructions. The analyses were performed under an air flow rate of 200 μmol s^−1^ at 25 °C, 65% humidity, 350 μmol (photon)·m^−2^·s^−1^ light intensity, and 350 μmol·mol^−1^ ambient CO_2_ concentration. The measurements were performed at 13:00~14:00 h local time. Five samples from each treatment were used for the analysis.

### Chlorophyll fluorescence determination

The chlorophyll fluorescence parameters were measured with a pulse-amplitude-modulated (PAM-2500) fluorometer (Walz, German). The leaves from each treatment were subjected to dark adaptation for more than 20 min. After the dark adaptation, the *F*o and *F*m values were measured under low modulated light over a period of 0.8 s. The maximum fluorescence in the light-adapted state (*F*m’) was recorded after a second saturation pulse. The actinic light (7,000 μmol m^−2^ s^−1^) was then turned off, and the far-red light was turned on to measure the minimal fluorescence in a light-adapted state (*F*′o). The *F*v/*F*m, *ΦPSII*, and *NPQ* values were calculated as follows: (*F*m − *F*o)/*F*m, (*F*m′ − *F*′)/*F*m′ and *F*m/*F*m′ − 1, respectively^51–53^.

### Extraction and assay of photosynthetic enzymes

The 100 mg frozen leaf samples were ground using a chilled mortar and pestle with 1 mL of extraction buffer.

An aliquot of the homogenate was collected to determine the total chlorophyll content in 96% ethanol, the remainder was centrifuged at 20,000 × g at 4 °C for 10 min, and the supernatant was used immediately for the enzyme activity assay.

To extract of NADP-dependent malate dehydrogenase (NADP-MDH) (EC 1.1.1.82) and NADP-malic enzyme (NADP-ME) (EC 1.1.1.40), extraction medium containing 50 mM sodium acetate (pH 6.0), 1 mg mL^−1^ bovine serum albumin (BSA), 0.04 mM phenylmethylsulfonyl fluoride (PMSF), 4 mM dithiothreitol (DTT), and 0.1% (v/v) Triton X-100 was used. NADP-MDH activity was assayed as described by Scheibe (1988)^54^. The reaction mixture (1 mL) contained 100 mM Tris-HCl (pH 8.0), 1 mM EDTA (pH 7.6), 0.2 mM NADPH, 1 mM DTT, 0.1 mg mL^−1^ BSA, and 50 μL of crude extract. The reaction was initiated by adding 50 μL of 40 mM oxaloacetate to the assay medium. To measure the NADP-ME activity, a method described by Kanai and Edwards (1973) with a slight modification was employed^55^. The reaction mixture (1 mL) contained 50 mM HEPES-KOH (pH 8.0), 20 mM MgCl_2_, 2.5 mM EDTA (pH 7.6), 0.5 mM NADP, 5 mM DTT, 0.05% (v/v) Triton X-100, and 50 μL of crude extract. The reaction was initiated by adding 50 μL of 0.1 M L-malate to a final concentration of 5 mM.

To extraction phosphoenolpyruvate carboxylase (PEPCase) (EC 4.1.1.31) and ribulose-1,5-bisphosphate carboxylase (RuBPCase) (EC 4.1.1.39), extraction medium containing 50 mM HEPES-KOH (pH 7.8), 5 mM MgCl_2_, 1 mM EDTA (pH 7.6), 5 mM DTT, and 1 mM PMSF was used. The PEPCase activity was assayed according to Omoto *et al*.^56^. The reaction mixture contained 50 mM HEPES-KOH (pH 8.0), 5 mM NaHCO_3_, 10 mM MgCl_2_, 0.1 mM NADH, 5 U mL^−1^ NADH-MDH, 5 mM DTT, and 50 μL of crude extract. The reaction was initiated by adding 50 μL of 0.1 M phosphoenolpyruvate. The RuBPCase activity was assayed according to Du *et al*.^57^. The reaction mixture contained 50 mM HEPES-KOH (pH 8.0), 10 mM NaHCO_3_, 20 mM MgCl_2_, 0.2 mM NADH, 2.5 mM ATP, 10 mM KCl, 1 mM EDTA (pH 7.6), 5 mM DTT, 5 mM phosphocreatine, 6 U mL^−1^ 3-phosphoglyceric phosphokinase and glyceraldehyde-3-phosphate dehydrogenase, 20 U mL^−1^ phosphocreatine kinase, and 50 μL of crude extract. The reaction was initiated by adding 50 μL of 12 mM RuBP.

To extract pyruvate orthophosphate dikinase (PPDK) (EC 2.7.9.1), extraction medium containing 50 mM HEPES-KOH (pH 7.4), 10 mM MgCl_2_, 1 mM EDTA (pH 7.0), 10% (v/v) glycerol, 5% (w/v) polyvinylpyrrolidone, 5 mM DTT, 0.01 mM leupeptin, 2 mM sodium pyruvate, and 2 mM KH_2_PO_4_ (pH 8.0) was used. The PPDK activity was assayed according to Fukayama *et al*.^58^. The reaction mixture contained 50 mM HEPES-KOH (pH 8.0), 10 mM NaHCO_3_, 10 mM MgCl_2_, 5 mM NH_4_Cl, 5 mM glucose-6-phosphate, 10 mM DTT, 2.5 mM KH_2_PO_4_ (pH 8.0), 2 mM sodium pyruvate, 0.2 mM NADH in 50 mM Tris-HCl (pH 7.4), 0.1 mM EDTA (pH 7.0), 12 U mL^−1^ NADH-MDH, 1 U mL^−1^ PEPCase in 20% (v/v) glycerol and 50 mM Tris-HCl (pH 7.5), and 50 µL of crude extract.

The reaction was initiated by adding 25 μL of 50 mM ATP (pH 7.0).

These enzyme activities were measured spectrophotometrically at 340 nm by monitoring changes in the absorbance, and they were expressed on a chlorophyll basis.

### Statistical analysis

The experiment was conducted in a randomized complete block design. The data were analysed using Statistical Package for the Social Sciences (SPSS) software version 17.0, and all of the values are presented as the mean ± SE. The means were separated using the least significant difference (LSD) test at the 5% probability level. The use of “difference” between treatments implies a significant difference (P = 0.05), whereas “no difference” implies no significant difference.
